# The experience of vivid autobiographical reminiscence is supported by subjective content representations in the precuneus

**DOI:** 10.1038/s41598-018-32879-0

**Published:** 2018-10-08

**Authors:** Vishnu Sreekumar, Dylan M. Nielson, Troy A. Smith, Simon J. Dennis, Per B. Sederberg

**Affiliations:** 1 Department of Psychology, The Ohio State University, Columbus, OH USA; 2 Department of Psychological Science, University of North Georgia, Oakwood, GA 30566 USA; 3 School of Psychology, University of Melbourne, Melbourne, VIC Australia; 4 Present Address: Surgical Neurology Branch, NINDS, National Institutes of Health, Bethesda, MD 20892 USA; 5 Present Address: Data Science and Sharing Team, Section on Functional Imaging Methods, Laboratory of Brain and Cognition, NIMH, National Institutes of Health, Bethesda, MD 20892 USA; 6 Present Address: Department of Psychology, University of Virginia, Charlottesville, VA 22903 USA

## Abstract

The human posteromedial cortex, which includes core regions of the default mode network (DMN), is thought to play an important role in episodic memory. However, the nature and functional role of representations in these brain regions remain unspecified. Nine participants (all female) wore smartphone devices to record episodes from their daily lives for multiple weeks, each night indicating the personally-salient attributes of each episode. Participants then relived their experiences in an fMRI scanner cued by images from their own lives. Representational Similarity Analysis revealed a broad network, including parts of the DMN, that represented personal semantics during autobiographical reminiscence. Within this network, activity in the right precuneus reflected more detailed representations of subjective contents during vivid relative to non-vivid, recollection. Our results suggest a more specific mechanism underlying the phenomenology of vivid autobiographical reminiscence, supported by rich subjective content representations in the precuneus, a hub of the DMN previously implicated in metacognitive evaluations during memory retrieval.

## Introduction

Tulving^1,2^ suggested that episodic memory is a unique human capability that enables us to engage in mental-time travel along a subjective timeline to reinstate past experiences. In a previous study, we identified the neural correlates of the *objective* spatiotemporal axes along which mental travel occurs during autobiographical memory retrieval^3^. However, the concept of episodic memory is incomplete without a notion of the self, the accompanying subjective dimensions of experience, and a special inwardly turned state of consciousness–termed autonoetic awareness–that guides retrieval and monitoring of autobiographical memories. In this paper, we describe the networks involved in representing *subjective*, self-relevant content of real-world events during autobiographical reminiscence.

Autobiographical memory concerns our personal histories and encompasses both episodic and personal semantic memory^4,5^. For example, knowledge about “I play ultimate frisbee every Wednesday” is part of autobiographical memory, but it need not necessarily be accompanied by a specific episodic memory or vivid recollection of the details surrounding a particular instance of having played ultimate frisbee. This type of personal semantics, operationalized as autobiographical knowledge or information extracted from repeated autobiographical events, has recently garnered a lot of attention and is thought to be an intermediate entity between semantic and episodic memory^6^. The recollective experience results only when details of a specific event are reinstated^5,6^. Therefore, everyday acts of memory involve guidance by retrieval of personal semantic knowledge culminating in the retrieval of a specific episode^7–9^. Additionally, vivid reminiscence is a hallmark of episodic recollection^10,11^ and therefore, in this study, we investigate the brain networks that subserve personal semantics and identify the specific parts of these networks that support the phenomenological experience of vivid autobiographical memory.

Given the special status of the self in autobiographical memory, it is likely to engage brain networks that have previously been found to be involved in processing information in relation to the self^12^. Specifically, the default mode network (DMN)^13,14^ has been associated with internally oriented processing across domains like memory^15–19^, prospection^20–22^, mental imagery^15^, and mind-wandering^23^. Consistent with this general conception of the DMN, an emerging body of neuroimaging work suggests that the human posteromedial cortex, which includes core regions of the DMN such as the retrosplenial cortex, posterior cingulate cortex (PCC) and the precuneus, is involved in episodic memory^24–30^. Recently, attempts have been made to characterize the various subsystems of the DMN. For example, a dual subsystems view of the DMN was proposed^31^ where midline cortical regions including the medial prefrontal cortex and PCC/precuneus were hypothesized to be associated with self-referential processing^32^, whereas the more lateral regions such as the medial temporal lobe (MTL) were thought to be involved in episodic retrieval. However, it is not clear what is represented or processed in the DMN during “self-referential processing”. Some prior work has investigated levels of activity recruited by personalized image cues versus generic cues^33^ but their results do not speak to multivariate representations of content. It is also not known if retrieving memories of real-world experiences spanning several weeks using highly personalised visual memory cues utilizes the same networks previously identified using generic memory cues in order to represent the content of retrieved memories (e.g. it has been argued that the observation of a left-lateralized parietal retrieval network could be a result of the limited range of verbal memory cues used in previous studies^34^). Whereas previous studies compared retrieval of controlled autobiographical memories of pictures taken on campus with retrieval of laboratory events^35^, the current study focuses on naturally occurring autobiographical events extending over much longer spatiotemporal scales with richer personally-relevant attributes. Recent studies have employed wearable cameras to investigate distributed brain activity patterns during memory retrieval^36,37^ but they focused on classifying mnemonic output (e.g. remember vs familiar vs new) rather than representational content. Therefore, critical questions remain about the specific functional roles and information content of the various regions of the recollection network^38^, particularly in a relatively more ecologically valid autobiographical reminiscence task. Critically, we had access to participant-generated content labels for each recorded episode from their lives which allowed us to track specific representations of personal semantics across each individual’s brain as they relived their experiences cued by images chosen from their own lives.

In a previous study focused on the MTL, we found that the anterior hippocampus represents objective space and time content, i.e., the “where” and “when” during retrieval of autobiographical memory extending over spatiotemporal scales of up to 30 Km and 1 month^3^. In the current paper, we perform multivariate pattern analysis on activity across the whole brain to investigate the brain networks that subserve subjective contents (i.e., the “what”) of AM and identify the specific parts of these networks that support the phenomenological experience of vivid autobiographical memory recollection.

## Methods

### Participants

Participants were recruited using advertisements placed on notice boards in multiple buildings on the main campus of The Ohio State University. To join the study, potential participants had to be willing to participate in the lifelogging data collection and to be willing and able to undergo an MRI scan. They were compensated at the rate of $10 per day for wearing the smartphone to collect data and at the rate of $15 per hour for the fMRI session. We recruited 10 participants (aged 19–26 y, mean age = 21.4 y; nine female), nine of whom wore the smartphone for ~1 month. The tenth participant wore the smartphone for 2 weeks. One participant (male) did not complete the fMRI session due to discomfort in the scanner; therefore, we did not include the data for that participant in any of our analyses. We collected an average of 5414 ± 578 SEM images per participant. These data were initially collected and analyzed for a previous publication focused on the representation of objective space and time in the MTL^3^. Therefore, the task is episodic in nature in the current study as well but the whole-brain multivariate analysis here probes the representation of personal semantic labels of experienced real-world events.

Our study has a similar number of participants as other fMRI studies using lifelogging devices (e.g. 13 participants and 10 days of lifelogging^39^; 10 participants and 2 days of lifelogging and a 5 month follow-up^40^). Another group, in two studies^36,37^, recruited 16 and 18 participants respectively but only included passive lifelogging (i.e., no additional effort required from participants other than wearing the device). Another study we know of with an active component to the lifelogging by asking participants to provide additional information about events at the end of each day had 23 participants but only included 6 days of lifelogging and 12 events from each day tested in the fMRI scanner^41^. In contrast, participants in the current study engaged in active lifelogging (see description of the end-of-day task later in this section for details) for ~1 month and relived 120 events from their lives in the fMRI scanner, covering a broader spatiotemporal extent of experience. Furthermore, we present individual plots for the main result and show that the effect exists at the level of every individual participant, mitigating some concerns about the sample size.

### Ethics Statement

The research protocol was reviewed and approved by the Institutional Review Board at The Ohio State University. Written informed consent was obtained from all participants, once before the lifelogging data collection phase and once before the fMRI session. All study procedures were performed in accordance with relevant guidelines and regulations.

### Device and Software

**Figure 1:**
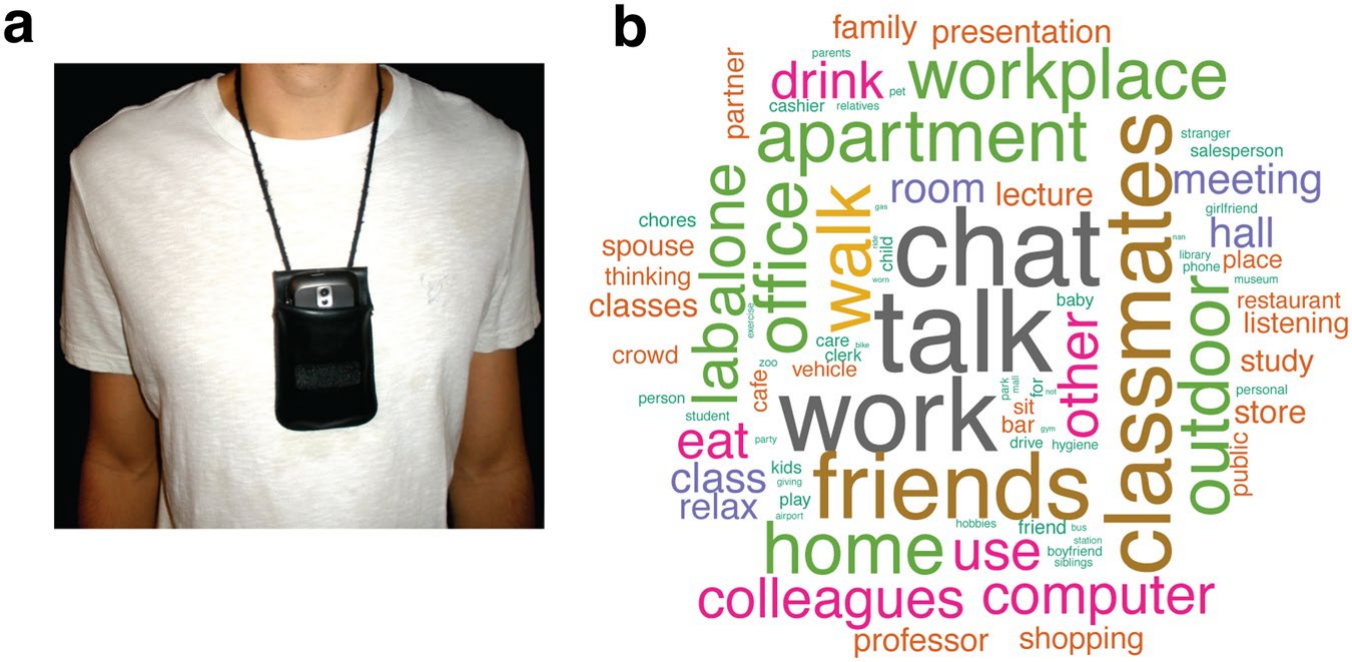
(**a**) The phone is worn around the neck with its camera exposed as shown. (**b**) A word cloud of the tags associated with the stimuli used in the fMRI experiment across all participants. Relative font sizes indicate relative frequencies of the tags while color and orientation are merely for visualization purposes.

Each participant carried an Android-based smartphone in a pouch attached to a neck strap as shown in Fig. 1a from morning until evening. The smartphone was equipped with a custom lifelogging application that acquired image, time, audio (obfuscated), GPS, accelerometer, and orientation information throughout the day and uploaded those data to a secure remote server when the smartphone was connected to a charger and detected WiFi. This transmission usually happened once per day at the end of the day because users charged the phone overnight. The data were sent in batch mode via SFTP (Secure File Transfer Protocol) for added security and remained inaccessible to other users in the system. The participants had control over what data they wanted to share with the experimenters. They were instructed on how to delete data from the phone and from the server. They were also allowed to turn the application off or to place a flap over the camera lens at any time during the data collection period when they felt the need for privacy. The lifelogging application was written by our programmers using Java (Oracle Corporation) to run in the background as a service. Data acquisition times could be fixed or variable, and they were determined by a movement based trigger to preserve battery resources when the user was not very active.

### Experimental Design and Statistical Analysis

#### Behavioral tasks

There were two main behavioral tasks that were performed before the MRI session. The first behavioral task was performed each evening during the lifelogging period. After the smartphone was connected to a power outlet to be charged overnight and had uploaded the data to our server, participants reviewed the images from that day through a web interface, a link to which was uniquely generated for each participant and provided to the participant before data collection, segmenting their stream of images into distinct episodes and tagging each episode with a set of tags chosen from a drop-down menu (Table 1). This master list of tags was constructed based on pilot studies we ran prior to the current study both on ourselves as well as with students in a large undergraduate class which is a good representation of the population from which participants in the current study were recruited. Participants were instructed to choose tags that best captured the contents of that episode and those that were likely to be good memory cues. The tags belonged to one of three categories: places, activities, and people but participants were free to choose any number of tags from any number of categories. If no tag fit the episode, participants could choose “other”. For each episode, they also provided a brief title and description. Insofar as only the participant knew the right tag to pick for a given episode, the set of tags captures the subjective contents of that episode. For instance, looking at someone else’s data with images of a person in it, it may be difficult to pick the appropriate tag from amongst “Spouse/Partner”, “Boyfriend/Girlfriend”, “Family”, “Work colleagues”, “Stranger”, and “Friends/Classmates”. While other tags are more objective, such as “Salesperson/Clerk/Cashier” or “Gas station”, the chosen tags are nevertheless the aspects chosen by the participant as the most salient of that episode from potentially many other descriptors. Therefore, the current analyses which are based on participant-generated content tags capture more self-relevant and subjective aspects of experience than did our previous work^3^ which based on objective GPS locations and timestamps. A word cloud of the tags belonging to the episodes used in the fMRI experiment across all nine participants is shown in Fig. 1b. The second behavioral task was conducted midway through the lifelogging period and at the end of the lifelogging period. After they collected data for two (and/or four) weeks, participants came into the laboratory on the Thursday of the third (and/or fifth) week and were tested over their ability to identify when events depicted in images drawn from his/her own lifelogs occurred. Specifically, they were shown a series of images from the weekdays of the preceding 2 weeks on the computer screen one at a time and asked to determine whether the image was from the first week or the second week. The results of this week discrimination task will be reported in a separate paper.

**Table 1:** The 51 tags available to participants across three categories: places, activities, and people. The number of available tags in each category are in brackets. Additionally, they could also choose “other” if none of these fit the event.

Category	Tags
Places (16)	Outdoor, Airport/Bus-station, Gas station, Park/Museum/Zoo, Gym, Library, Parents’/siblings’/relatives’ home or apartment, Mall, Friend’s home/apartment, Class/meeting room/hall, Restaurant/Cafe/Bar, My office/lab/workplace, Home/apartment, Other office, Store, Other person’s office/workplace
Activities (22)	Chores, Thinking, Party, Talk on phone, Use a computer, Exercise, Shopping, Personal hygiene, Relax, Eat/drink, Talk/chat with other(s), Phone not worn, Study, Work, Drive, Care for/play with child/baby, Ride bike, Giving a lecture/presentation, Listening to a lecture/presentation, Walk, Sit in a vehicle, Hobbies
People (13)	Kids, Family, Friends/Classmates, Pet, Salesperson/Clerk/Cashier, Boyfriend/Girlfriend, Stranger, Alone, Professor (of my classes), Student, Spouse/Partner, Crowd (in a public place), Work colleagues

#### Analysis of tag co-occurrence structure

In order to characterize the co-occurrence structure of semantic tags that emerges across participants, we computed pointwise mutual information (PMI), a measure of association between two features. PMI for a pair of tags *x* and *y* is given by:

1 $$PMI(x,y)=lo{g}_{2}\frac{P(x,y)}{P(x)P(y)}.$$

The probabilities in Eq. 1 are calculated by accumulating frequencies of tags as well as frequencies of co-occurrences of tag pairs in all events across participants and then dividing by the total number of events (120 × 9 = 1080). PMI is sensitive to tag frequency and is bounded between −∞ and min[−*log*_2_*p*(*x*), −*log*_2_*p*(*y*)]. Therefore, we used the normalized pointwise mutual information (NPMI) which is more easily interpretable and is less sensitive to tag frequency:

2 $$NPMI(x,y)=\frac{PMI(x,y)}{h(x,y)},$$

where

3 $$h(x,y)=-\,lo{g}_{2}p(x,y).$$

*NPMI*(*x*, *y*) = −1 when the pair of tags never co-occurs, *NPMI*(*x*, *y*) = 0 indicates that the tag occurrences are independent of each other, and *NPMI*(*x*, *y*) = 1 indicates that the tags always co-occur.

#### MRI acquisition

MRI data were acquired on a 3-T Siemens Magnetom Trio TIM system with a 16-channel head coil. Anatomical images were acquired with a sagittal, T1-weighted, magnetization prepared rapid acquisition gradient echo sequence [1.0-mm isotropic voxels, repetition time (TR) = 1900 ms, echo time (TE) = 4.68 ms, 160 slices with field of view (FoV) = 256 mm]. Functional images were acquired with an echoplanar imaging sequence (2.5-mm isotropic voxels, TR = 3000 ms, TE = 28 ms, flip angle = 80, 47 slices with FoV = 250 mm).

#### Stimuli selection

We selected 120 images from each subject’s lifelogging data to present to the subject in the scanner. First, we excluded pictures of floors/ceilings/walls, blurry images, and images with inadequate exposure. Then, we selected images that appeared to have enough detail that they could act as cues for distinct episodes. From this subset of images, we selected images representing events that spanned the entire period each participant wore the lifelogging device, with as uniform sampling of events as possible.

#### Image representations

To control for visual similarity in our analyses, we used the common neighbor ratio measure that we introduced previously^42^, in order to compare and pick one representation from amongst five different image representations popular in computer vision. Visual dissimilarities between image stimuli were then calculated based on the selected image representation and were entered as covariates in the general linear models relating BOLD pattern dissimilarities to dissimilarities between sets of personal semantic tags and vividness judgments (described later).

All images were resized to 640 × 480. The color histogram and color correlogram^43^ representations were computed as in^42^. Additionally, we explored Histogram of Oriented Gradients (HOG^44,45^, GIST^46^, and Speeded-up Robust Features (SURF)^47^. SURF is a faster version of the Scale Invariant Feature Transform (SIFT)^48^. We provide brief high-level descriptions of each representation in the Supplementary Information available online and ask that readers refer to the original papers for more details.

#### Comparing image representations: Common neighbor ratio

**Figure 2:**
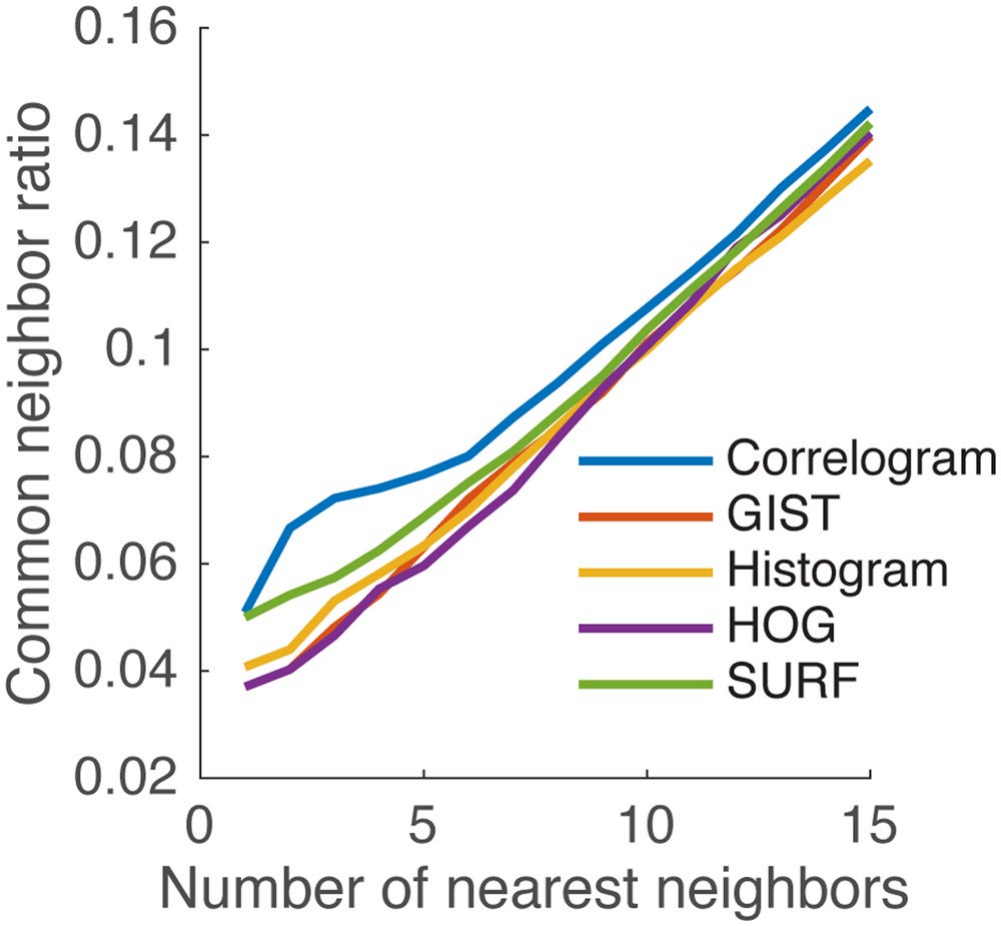
Common neighbor ratio comparison of image representations. The color correlogram representation achieves the best congruence between visual and temporal proximity of nearest neighbors (k).

For each image representation, visual dissimilarity between image pairs was computed as the Euclidean distance between normalized reduced-dimensional image vectors^42^. To pick the “best” image representation for subsequent analyses, we required that our representation of choice and the associated distance measure accurately identify images from similar contexts as being similar to each other. Additionally, in general, episodes that are close in time tend to occur in similar spatial contexts (e.g. walking from one room to the next in an office) and hence should be identified as being visually similar. Also, while we did attempt to choose stimuli that did not come from the same episode, people do go back to the same spatial locations multiple times within a day^42^. With this in mind, we defined the common neighbor ratio (CNR)^42^. Given a positive integer *k*, for each image *I*, we find its *k* nearest neighbors both in the visual domain and in the time domain. Suppose ${D}_{I}=\{{I}_{d1},{I}_{d2},{I}_{d3},\ldots \,,{I}_{dk}\}$ are image *I*’s *k* nearest neighbors in space and ${T}_{I}=\{{I}_{t1},{I}_{t2},{I}_{t3},\ldots \,,{I}_{tk}\}$ are image *I*’s *k* nearest neighbors in time, then

4 $$CNR=\frac{\sum _{I=1}^{n}|{D}_{I}\cap {T}_{I}|}{nk}$$

where *n* is the total number of images (we performed this on the 120 image stimuli for each participant). If *k* equals *n* − 1 (i.e., all the other images in the set), then the ratio is 1. The method that has a higher common neighbor ratio is the better one for our purpose, which is to successfully identify images that came from proximal temporal contexts as similar. Figure 2 shows common neighbor ratios averaged over participants for each image representation. For all reasonable values of *k* nearest neighbors (given that care was exercised while selecting stimuli to avoid images that came from the same episode too often, it is unlikely that there are many neighbors from the same temporal context in any given stimulus set of 120 images and so we explored values of *k* up to 15), we found that the color correlogram achieves better congruence between visual and temporal proximity and hence chose the color correlogram as our preferred image representation.

#### fMRI Experiment

**Figure 3:**
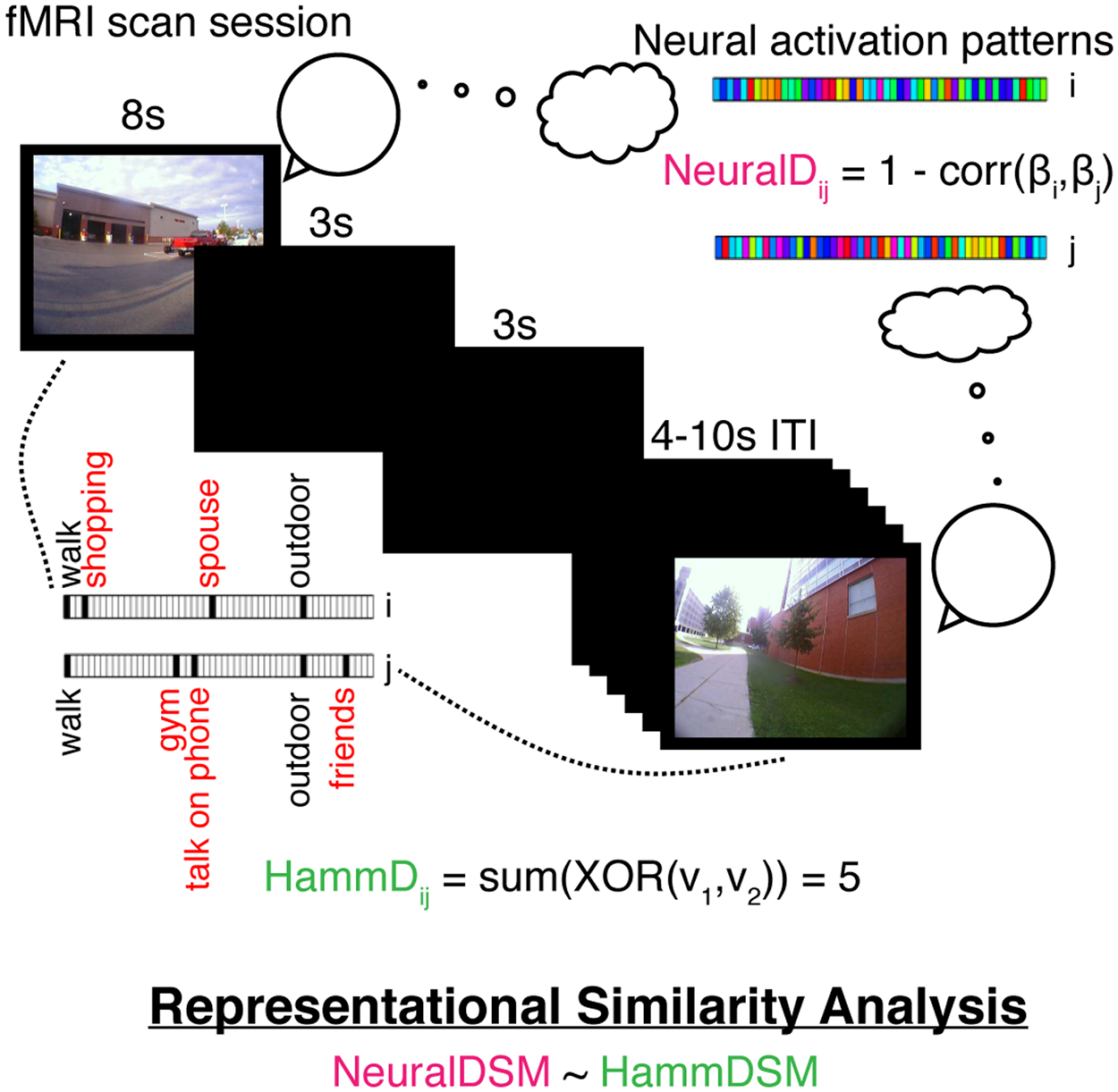
Depiction of the fMRI experiment and Representational Similarity Analysis (RSA). Participants are shown images from their own lives and are instructed to relive the associated experiences. The neural activity during this reminiscence period is analyzed using RSA to investigate whether distances between neural patterns (NeuralD_ij_) corresponding to pairs of image cues (an example of such a pair is shown) relate to distances between the corresponding sets of semantic tags (HammD_ij_). After the reminiscence period, participants indicate whether they remember the event and then report the vividness of their recollective experience.

In the scanner, participants were instructed that they would be viewing images from the experience sampling experiment they recently completed and told that each image would be displayed for 8 s. Participants were asked to “… try to remember the event depicted in the picture, and try to relive your experience mentally”. After the remembrance period for each event, participants were asked if they remembered the event (“yes” or “no”) and how vividly they recalled the event (“lots of detail” or “very little detail”). Participants were given 2.5 s to respond to each of those questions using a button box held in their right hand. The images were presented in random order, and the task was split into eight runs with 15 images in each run. With each image presented for 8 s and each question for presented 2.5 s with a 0.5 s interstimulus interval, each trial took a total of 14 s. The intertrial interval was jittered uniformly between 4 and 10 s, allowing for a true event-related design. Figure 3 summarizes the fMRI experimental design and representational similarity analysis.

#### fMRI Processing

fMRI processing was carried out using Analysis of Functional NeuroImages (AFNI)^49^ and Functional Magnetic Resonance Imaging of the Brain (FMRIB) Software Library (FSL)^50^. The T1-weighted anatomical image was intensity-normalized, skull-stripped, and warped to a 2.5-mm MNI-152 template using *3dQwarp*. We selected a 2.5 mm template to match the resolution of the functional scans. For the functional scans, we dropped the first two TRs of each run, then removed spikes with *3ddespike* and temporally shifted all of the slices in each volume to the start of the TR using *3dTshift* with Fourier interpolation. We then warped the functional scans to template space, blurred them to 4 mm FWHM using *3dBlurtoFWHM*, and scaled the voxel values to a mean of 100 (maximum of 200) for each run. At this point, we performed independent component analysis of each functional run with FSL’s *MELODIC*. Components were visually inspected to identify noise components following published guidelines^51^. Noise components were regressed out of the functional runs using FSL’s *fsl_regfilt* command. We then ran a regression with restricted maximum likelihood estimation of temporal autocorrelation structure on the filtered functional runs using *3dDeconvolve* and *3dREMLfit* to generate single-trial betas for each reminiscence trial and to regress out the effects of the mean and derivative of motion terms, as well as cerebrospinal fluid signal. The regressor for each image presentation was an 8-s block convolved with a hemodynamic response function. The neural activity of the question prompts were accounted for with a 2.5 s block convolved with a hemodynamic response function. We modeled response processing and motor activity related to the button push with a set of nine tent functions (piecewise linear splines; see^52^) over the 16 s after the question response. Including these tent functions in our model allowed us to estimate the motor response robustly for each subject so that the signal from the motor responses did not contaminate the single-trial beta fit for each reminiscence period. Lastly, we regressed out local white matter signal with *3dAnaticor*. Researchers were not blinded during preprocessing or subsequent analyses.

#### Representational similarity analysis

Representational Similarity Analysis (RSA^53^) is a data-analytic framework that allows us to quantify the relationship between the multivoxel patterns of neural activity and the behavior of interest. We used RSA to predict dissimilarities between the neural representations of events based on the dissimilarities between the events in terms of their subjective contents as captured by the tags provided by participants during the lifelogging phase as well as the vividness ratings provided during the reminiscence task in the scanner. See Fig. 3 for a depiction of the task and analysis. The basic logic of the analysis is as follows: autobiographical recollection comprises personal semantics as well as recollection of details (i.e., vivid reminiscence). We first search over the whole brain for regions that represent personal semantics in general. We then use the vividness ratings in the data to characterize the network that represents personal semantics during vivid reminiscence. However, in this second analysis, the regions discovered may include some that represent personal semantics during both vivid and non-vivid reminscence. Therefore, in a third analysis, we look for regions that not only represent personal semantics during vivid reminiscence but those that do so to a greater extent during vivid compared to non-vivid reminiscence.

For each pair of images presented to the participants, we calculated the Hamming distance between the associated tag sets. Since a total of 52 unique tags were used (including the “other” tag), each tag set can be represented as a 52-dimensional binary vector where each entry denotes the presence/absence of a tag. The Hamming distance between two binary vectors A and B is simply the number of positions where they differ, or in other words, Hamming distance = sum(XOR(A, B)). For example, if A = [1 1 0 0 1 0 1 …] and B = [0 0 1 1 0 0 1 …] with only the first 5 positions being different, the Hamming distance is 5. As a more concrete example, if image A had been tagged with Walk, Outdoor, Talk on phone and image B had the tags Walk, Store, Talk on phone, the Hamming distance between them is 2 since there are 2 tags that are different between the two sets reflecting the difference in location between the two otherwise similar events.

In our previous analysis^3^, for each pair of images presented to the participants, we calculated the geodesic distance in meters between the two GPS coordinates and the difference in time in seconds. Geodesic distance was calculated using the GeoPy Python package. Image pairs with spatial distances less than 100 m were excluded because these distances are below the reliability of the GPS radios in these smartphones. Image pairs with temporal distances below 15.6 h were excluded based on prior work because of a discontinuity in the spatiotemporal distribution of image pairs^42^. The discontinuity between 14 and 16-h results from participants taking off their cameras to sleep. This gap is propagated through the rest of the results as a relative lack of image pairs that are multiples of ~15 h apart. An analysis of the structure of similar lifelogged images demonstrated that image pairs taken from identical spatiotemporal locations occupied a lower dimensional manifold than those image pairs taken from separate spatiotemporal locations^42,54^. By removing image pairs separated by less than 100 m and 15.6 h, we reduced the possibility that the images themselves would give rise to the present results as a consequence of within- and between- episode image properties. Some participants spent time out of town during the period of data collection, resulting in a small portion of image pairs with spatial distances greater than 30 km; these image pairs were also excluded in^3^ and we impose the same spatial limit. Images that were blurry or contained reflections of the participants were also excluded. Hamming distances between the tag sets of the remaining pairs of image stimuli were calculated as described earlier. In order to further control for visual similarity, we compared five different popular image representations based on how well they identified temporally close images as visually similar and chose the color correlogram representation^43^. Euclidean distances between the correlogram image representations were computed and entered into the General Linear Models (GLMs) described below as a visual control (*VisSim* in Eqs 5 and 6).

In order to investigate both cortical and sub-cortical contributions to content retrieval, we performed a whole-brain searchlight analysis^55^ using the PyMVPA package^56^. Representational similarity analysis (RSA) was performed on voxels within spherical neighborhoods of 7.5 mm radius surrounding a central voxel. An initial 2.5 mm resolution gray matter mask in the Montreal Neurological Institute (MNI-152) standard space was used to input the fMRI data to the searchlight function but for each individual, we used a subject-level gray matter mask warped to MNI-152 space to select the spheres on which to run the analysis. Within each sphere, the neural distance for each image pair was calculated as 1 minus the Pearson correlation between the voxel-level single-trial betas for the trials corresponding to those image pairs. Neural distances were z-scored within participants since individual differences in BOLD activation levels can lead to differences in the scale of neural distances. Z-scoring provides a way to put the model coefficients estimated within individuals on an equal footing for a group analysis. In each searchlight sphere in each subject, we ran the following GLM:

5 $$neuraldistance=\alpha +{\beta }_{Hamm}Hamm+{\beta }_{VisSim}VisSim+{\beta }_{scanner}lo{g}_{10}(scannertime)+\varepsilon $$

*Hamm* are the Hamming distances between pairs of tag sets as described earlier. Therefore, *β*_*Hamm*_ is the term we are interested in here in order to specify how neural activity patterns correspond to relationships between personal semantic representations. *VisSim* are the distances between image representations. Scanner time was calculated as the number of seconds between presentation of the images during the fMRI experiment. We used the log of time based on previous literature that has shown a power-law relationship for neural representations^57^. In each sphere, we performed a t-test on the betas from the subject-level GLMs to determine if they were significantly different from zero across participants. We used nonparametric permutation to test for significance^58^ because the pairwise nature of the distances in our analysis violated the assumption of independent samples. Neural data were permuted with respect to behavioral data within participants. This process was repeated for 1000 permutations of the neural data. We performed threshold-free cluster enhancement (TFCE^59^) on the *Hamm* t-value maps for both the unpermuted data as well as for the 1000 permutations. The maximum and minimum TFCE values across all spheres for each permutation were recorded. The 97.5th percentile of the max TFCE values was chosen as the threshold above which a positive TFCE value in the unpermuted data is deemed to be significant. Similarly, we tested the negative end by using the 2.5th percentile of the min TFCE values as the threshold (this procedure is essentially a two-tailed test at p = 0.05). This analysis reveals the clusters of brain regions whose activity patterns reflect the relationships (captured by Hamming distances) between events in terms of their contents. Additionally, we wanted to identify regions that may support metacognitive judgments (such as vividness of the recollective experience) based on the contents of memory retrieval. One possibility is that the quality of personal semantic representations in such brain regions would differ between different levels of reported vividness. Therefore, we ran the following model to investigate the brain regions that represent subjective content during vivid but not during non-vivid reminiscence:

6 $$\begin{array}{rcl}neuraldistance & = & \alpha +{\beta }_{Hamm}Hamm+{\beta }_{Vivid}Vivid+{\beta }_{Hamm\ast Vivid}Hamm\ast Vivid\\  &  & +{\beta }_{VisSim}VisSim+{\beta }_{scanner}lo{g}_{10}(scannertime)+\varepsilon \end{array}$$

For a given pair of stimuli, *Vivid* was coded as 0 if both were reported to be vividly recalled, 0.5 if one of them was vivid, and 1 if neither was vivid. After subtracting out the effects of temporal proximity and visual similarity of the stimuli in the scanner, this coding scheme allows us to interpret *β*_*Hamm*_ as describing the relationship between Hamming distances and neural distances for vividly remembered events since the other terms vanish for *Vivid* = 0. While this analysis characterizes the network involved in representing subjective content during vivid reminiscence, it does not preclude regions that may also represent similar subjective content during non-vivid reminiscence, which is why we included the interaction term in the model. We expected the interaction between Hamming distance and vividness to be negative as that would indicate that the effect of Hamming distance is greater for vividly remembered events relative to less vividly remembered events in its ability to predict the neural distances between them. To identify the regions that show a significant effect of both Hamming distance by itself as well as the negative interaction with vividness, we performed a conjunction analysis by taking $Min({t}_{Hamm},-\,{t}_{Hamm\ast Vivid})$. TFCE was performed on this minimum t-statistic map and the permutation procedure was performed as earlier to assess significance of the clusters. 95th percentile of max TFCE across permutations was used to test significance since this was a one-tailed directional test. The conjunction analysis reveals the regions that reinstate subjective contextual details to a greater extent for vividly remembered events relative to the less vivid or non-vivid events.

Finally, to visualize the relationship between Hamming distances and neural distances in a sphere (radius = 7.5 mm) surrounding the peak voxel in the right precuneus, we used partial residual plots. Partial residual plots describe the relationship between a dependent variable and an independent variable after accounting for the contribution from other independent variables in a multivariate regression model. Specifically, to visualize the relationship between Hamming and neural distances for vividly remembered pairs of images (*Vivid* = 0 in Eq. 6), we first computed residuals by regressing neural distances versus all the independent variables in Eq. 6) for vivid pairs. *β*_*Hamm*_*Hamm* is then added to these residuals to get partial residuals = residuals + *β*_*Hamm*_. The partial residuals are plotted against *Hamm* to visualize the relationship between *Hamm* and neural distances for vivid pairs after taking into account the effect of all the other independent variables. This procedure can be understood intuitively if one considers the hypothetical case when all the other independent variables explain the response variable perfectly. In that case, residuals = −*β*_*Hamm*_*Hamm* and therefore the partial residuals after adding *β*_*Hamm*_*Hamm* back in would be 0. The regression lines overlaid on the partial residuals vs Hamming distance plot have the same slope as in the full model (i.e., *β*_*Hamm*_) but have an intercept of 0. Similarly, we plot the partial residual plot for the less vivid and non-vivid (*Vivid* ≠ 0) pairs, but now for components *β*_*Hamm*_*Hamm* + *β*_Hamm*Vivid_*Hamm* * *Vivid* since the relationship between Hamming distances and neural distances now also depends on *Vivid* via the interaction term (which was 0 for vivid pairs).

We also ran an analysis including the same regions of interest (ROIs) reported in our previous work^3^ (which were anterior hippocampus, middle hippocampus, posterior hippocampus, parahippocampal gyrus, and posterior V1 in both hemispheres for a total of 10 ROIs). We ran the two models in Eqs 5 and 6 as well as the same models with space, time, and their interactions included. In each ROI, we performed a t-test on the betas from the subject-level GLMs to determine if they were significantly different from zero across participants. Permutation tests (as described earlier) were then performed on these t-statistics.

## Results

### Behavioral Results

The Hamming distances between the tag sets for pairs of images across participants ranged from 0 to 15. 63 ± 5% SEM of the stimuli were reported as having produced successful reminiscence. The proportion of analyzed stimuli that were indicated as evoking vivid reminiscence by the nine participants ranged from 0.21 to 0.81 (mean 0.47 ± 0.07 SEM). The three different tag types were used to a similar extent across stimuli (Mean 95 ± 2% SEM of the stimuli contained activity tags, Mean 91 ± 4% SEM contained people tags, and Mean 95 ± 1% SEM contained place tags). No differences were apparent in the percentage of stimuli that evoked vivid reminiscence depending on the type of tag present (Mean 44.5 ± 5.4% SEM of the stimuli with activity tags, Mean 42.4 ± 5.1% SEM of the stimuli with people tags, and Mean 44.3 ± 5.7% SEM of the stimuli with place tags evoked vivid reminiscence) suggesting that vividness was not linked to the presence of any particular tag type.

**Figure 4:**
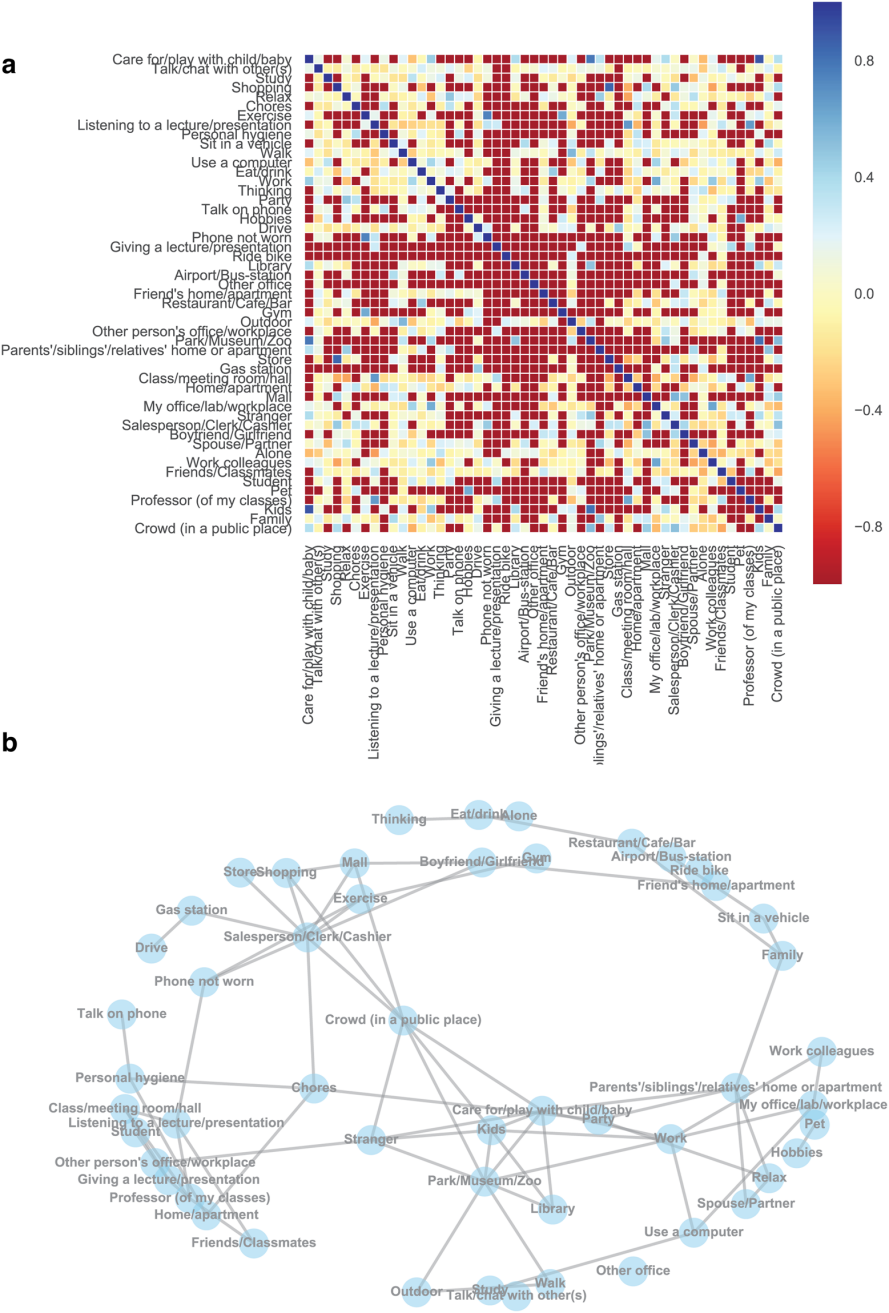
(**a**) Normalized pointwise mutual information (NPMI) between all pairs of tags, computed across participants. (**b**) A network of tags with *NPMI* > 0.2.

We visualized the co-occurrence structure of content tags provided by participants by computing normalized pointwise mutual information (NPMI) between pairs of tags (see Methods for details). NPMI ranges from −1 to 1, with −1 indicating that the pair of tags never occurred together, 0 indicating that the tag occurrences were independent of each other, and 1 indicating that the tags co-occurred perfectly with each other. The NPMI matrix is presented in Fig. 4a. We also plotted a network of tags with *NPMI* > 0.2 in Fig. 4b to visualize the co-occurrence structures that emerge across participants. The NPMI computations were performed across participants to get stable estimates of co-occurrence frequencies. Together, the panels in Fig. 4 demonstrate clusters surrounding university campus life and social/family life, reflecting general characteristics of the student pool from which we recruited participants.

### fMRI Results

**Figure 5:**
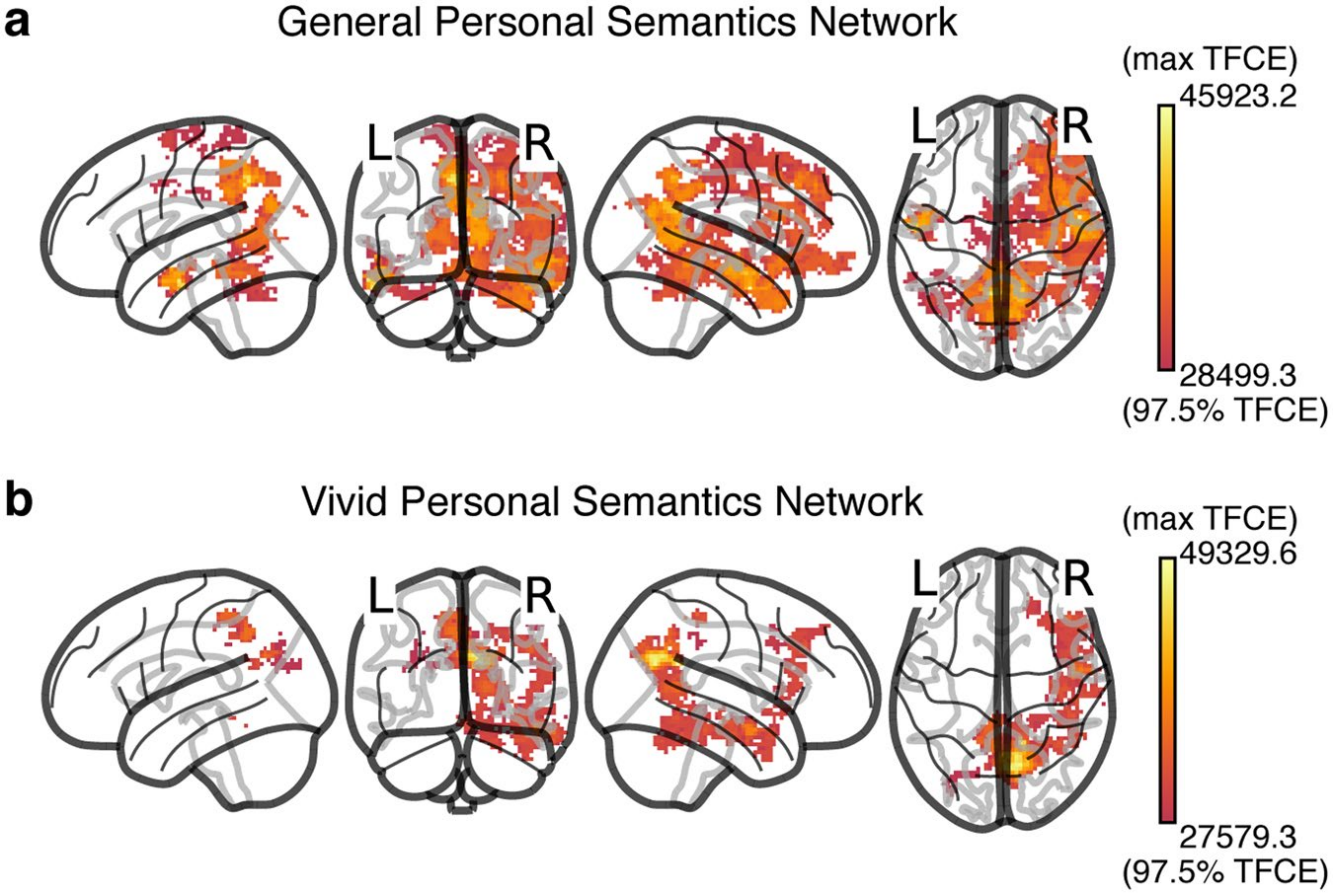
(**a**) The network of regions involved in the representation of general personal semantics as identified by the RSA analysis in Eq. 5 (corresponding to the *Hamm* term). Four different views (left, back, right, top) of a glass brain are shown. A full 3D view of the network can be seen in Supplementary Movie S1. See Table 2 for a complete list of regions with at least 10 voxels in the general personal semantic network. (**b**) The network of regions involved in the representation of personal semantics during vivid reminiscence as identified by the RSA analysis in Eq. 6 (corresponding to the *Hamm* term). See Table 3 for a complete list of regions with at least 10 voxels in the vivid personal semantics network. The same views presented in **(a)** are shown and comparing the two networks reveals that the vivid reminiscence network is a subset of the more general personal semantics network identified in (**a**). A full 3D view of the network can be seen in Supplementary Movie S2.

To reiterate the logic underlying the analysis, given that autobiographical recollection involves representation of both personal semantics as well as episodic detail accompanied by the phenomenology of vivid reminscence, we first ran a general linear model (GLM) to relate neural distances to distances between representations of personal, subjective content of the events. RSA with the model in Eq. 5 (a GLM relating neural distances with Hamming distances between tag sets) revealed a broad network of regions that represented personal semantics during the reminiscence task cued by participants’ own images. This general personal semantic network, shown in Fig. 5a, included core parts of the default mode network (DMN) such as the precuneus, anterior cingulate, posterior cingulate, middle temporal gyrus bilaterally, and a right lateralized network including the medial and prefrontal cortices, parts of the inferior parietal lobule (supramarginal and angular gyri), and the parahippocampal cortex (see Table 2 for a complete list of regions with at least 10 voxels in the network). A personal image cue can trigger memory for general facts about similar events, which need not lead to detailed and vivid reminiscence of a specific event. Because Eq. 5 did not include a term for vividness, *β*_*Hamm*_ tracks the regions that represent personal semantics generally and does not identify the regions that do so specifically during vivid reminiscence (implying greater episodic retrieval).

**Table 2:** Peak voxel coordinates of regions with at least 10 voxels in the general personal semantic network (Eq. 5, corresponding to the *Hamm* term, also see Fig. 5a). The FSL-Harvard-Oxford cortical-subcortical atlas was used to get coordinates in MNI space. When multiple sets of coordinates are shown for a region, they correspond to multiple peak voxels.

Region	Voxel count	Mean TFCE	Max TFCE^a^	MNI coordinates		
				*x*	*y*	*z*
R. Frontal Pole	544	30897.1	37065.1	35	39.5	40.5
L. Precuneus	469	33131.6	43831.9	−5	−53	43
R. Precuneus	375	34201.7	40640.8	12.5	−55.5	25.5
R. Middle Frontal Gyrus	334	31062.5	37003.5	27.5	29.5	40.5
R. Temporal Pole	327	32565.1	38100.2	45	7	−27
R. Precentral Gyrus	225	29847.2	33840.5	62.5	12	23
				57.5	9.5	18
R. Pos. Middle Temporal Gyrus	206	32870.3	39060.5	60	−10.5	−9.5
R. Pos. Cingulate Gyrus	200	32106.7	39004.5	15	−50.5	5.5
R. Superior Frontal Gyrus	161	30163.9	36520.4	25	29.5	45.5
R. Lingual Gyrus	154	31568.5	38727.5	12.5	−50.5	0.5
R. Postcentral Gyrus	149	29863.7	32535.1	60	−15.5	43
R. Inferior Frontal Gyrus, pars opercularis	132	31868.5	35433.1	52.5	17	20.5
R. Inferior Frontal Gyrus, pars triangularis	127	32038.2	35164.8	47.5	29.5	18
R. Pos. Superior Temporal Gyrus	123	32284.4	39818.7	47.5	−8	−17
L. Pos. Cingulate Gyrus	112	32028.4	37295.2	−2.5	−40.5	35.5
R. Supplementary Motor Cortex	111	29923.5	31504.1	10	7	55.5
L. Postcentral Gyrus	106	29940.4	36645.8	−2.5	−40.5	55.5
L. Ant. Middle Temporal Gyrus	102	33844.0	44029.8	−57.5	−10.5	−27
L. Lingual Gyrus	93	31611.5	35322.6	−10	−63	5.5
R. Frontal Orbital Cortex	91	31321.8	34199.9	40	19.5	−9.5
R. Insular Cortex	78	30916.3	34115.3	42.5	17	−7
				40	17	−7
				40	19.5	−7
R. Temporal Occipital Fusiform Cortex	76	31119.1	32987.4	37.5	−53	−12
				37.5	−50.5	−12
R. Ant. Parahippocampal Gyrus	75	30976.0	35190.0	27.5	2	−37
R. Middle Temporal Gyrus, temporooccipital part	73	28955.5	32818.4	50	−40.5	0.5
R. Ant. Middle Temporal Gyrus	68	33720.1	45923.2	52.5	−3	−27
L. Precentral Gyrus	67	29240.2	36004.3	−2.5	−35.5	55.5
R. Ant. Superior Temporal Gyrus	62	33084.1	39607.5	50	−0.5	−17
L. Inferior Temporal Gyrus, temporooccipital part	60	28585.0	29626.6	−52.5	−53	−14.5
R. Planum Temporale	55	31013.6	32529.0	57.5	−30.5	13
R. Angular Gyrus	52	30522.7	32509.5	60	−58	23
R. Ant. Supramarginal Gyrus	51	29566.7	31001.5	65	−28	40.5
R. Ant. Cingulate Gyrus	51	29465.8	32538.4	2.5	−3	33
L. Intracalcarine Cortex	50	32307.8	35476.6	−12.5	−63	5.5
R. Pos. Supramarginal Gyrus	50	29057.8	31393.8	50	−38	8
L. Pos. Middle Temporal Gyrus	46	30053.9	37805.2	−52.5	−10.5	−17
R. Cuneal Cortex	45	31713.7	38448.5	5	−68	20.5
R. Central Opercular Cortex	41	30675.2	32363.8	50	2	8
R. Intracalcarine Cortex	38	33532.0	38478.0	15	−60.5	5.5
L. Superior Frontal Gyrus	38	29254.3	30809.9	−7.5	−3	70.5
				−7.5	−3	73
				−7.5	−0.5	70.5
R. Planum Polare	36	30760.8	36536.2	45	−5.5	−17
L. Supplementary Motor Cortex	35	29523.5	30895.3	−2.5	−0.5	68
L. Ant. Cingulate Gyrus	31	28931.6	31007.8	−2.5	−3	33
R. Pos. Temporal Fusiform Cortex	31	31544.4	34842.5	40	−15.5	−29.5
L. Temporal Occipital Fusiform Cortex	25	29667.4	31681.3	−42.5	−55.5	−24.5
L. Ant. Superior Temporal Gyrus	24	34125.3	39511.7	−50	−8	−14.5
L. Planum Polare	23	29722.4	36573.1	−42.5	−0.5	−19.5
R. Pos. Inferior Temporal Gyrus	22	31040.6	32278.8	65	−25.5	−22
R. Pos. Parahippocampal Gyrus	19	30848.5	33924.4	15	−35.5	−12
				15	−33	−12
R. Supracalcarine Cortex	18	31093.0	35722.3	15.	−63.	13
				2.5	−68	15.5
R. Paracingulate Gyrus	16	28646.2	28939.7	5	22	48
L. Cuneal Cortex	15	30396.3	31544.9	−7.5	−88	23
L. Inf. Lateral Occipital Cortex	15	28542.3	28927.9	−45	−65.5	−9.5
L. Supracalcarine Cortex	13	31272.3	33972.7	−12.5	−65.5	13
L. Temporal Pole	13	29828.0	33602.5	−52.5	4.5	−22
L. Pos. Superior Temporal Gyrus	12	30542.7	39294.6	−55	−13	−7
R. Inferior Temporal Gyrus, temporooccipital part	11	31104.2	32365.4	47.5	−45.5	−27
R. Frontal Operculum Cortex	11	29880.5	31275.1	45	24.5	0.5
				45	24.5	3
				42.5	22	3

^a^97.5th percentile TFCE threshold = 28499.3, Max network TFCE = 45923.2.

In order to characterize the network that represents personal, subjective content during vivid reminiscence, we ran an RSA with Eq. 6, a GLM that included a vividness (*Vivid*) term for the overall level of vividness experienced when recalling events, as well as an interaction between Hamming distances (*Hamm*) and vividness. The main effect of *Hamm*, due to how *Vivid* was coded (see Methods), captured the relationship between neural distances and personal semantics for vividly reexperienced events. The interaction between *Hamm* and *Vivid* captured regions where neural distances tracked personal semantics to different extents across different levels of vividness. Therefore, the conjunction between *Hamm* and *Hamm* * *Vivid* tracked the regions that not only represent subjective content during vivid reminiscence but also show a decreased relationship between neural distances and personal semantic distances during less vivid reminiscence. Figure 5b shows the regions that represent personal semantic content during vivid reminiscence. This is mostly a sub-network of the general personal semantics network, but relatively more right lateralized and therefore this “vivid” personal semantic network also includes parts of the DMN, such as the precuneus bilaterally, and in the right hemisphere, posterior cingulate, parahippocampal cortex, medial and pre-frontal cortices (see Table 3 for a complete list of regions with at least 10 voxels in the vivid personal semantics network).

**Table 3:** Peak voxel coordinates of regions with at least 10 voxels in the vivid personal semantic network (Eq. 6, corresponding to the *Hamm* term, also see Fig. 5b). The FSL-Harvard-Oxford cortical-subcortical atlas was used to get coordinates in MNI space. When multiple sets of coordinates are shown for a region, they correspond to multiple peak voxels.

Region	Voxel count	Mean TFCE	Max TFCE^a^	MNI coordinates		
				*x*	*y*	*z*
R. Precuneus	382	34385.0	48123.3	12.5	−65.5	23
R. Middle Frontal Gyrus	193	30099.8	34687.5	45	32	33
L. Precuneus	160	31471.5	35883.0	−2.5	−63	28
R. Temporal Pole	91	30264.4	32481.3	50	12	−24.5
R. Lingual Gyrus	68	30309.1	32227.1	12.5	−58	5.5
R. Pos. Middle Temporal Gyrus	56	30272.1	31821.7	52.5	−18	−17
R. Cuneal Cortex	54	33206.4	45559.9	12.5	−68	23
R. Inferior Frontal Gyrus, pars triangularis	51	30391.2	31757.0	55	29.5	18
R. Temporal Occipital Fusiform	49	30347.1	32620.1	37.5	−50.5	−12
R. Pos. Cingulate Gyrus	46	30133.3	34881.8	12.5	−50.5	33
L. Sup. Lateral Occipital Cortex	45	27763.0	28185.4	−32.5	−83	20.5
R. Pos. Temporal Fusiform	37	30597.8	32950.2	42.5	−15.5	−24.5
R. Ant. Middle Temporal Gyrus	35	31463.4	37098.3	52.5	−3	−22
R. Pos. Inferior Temporal Gyrus	33	30424.2	33262.3	47.5	−33	−19.5
R. Ant. Superior Temporal Gyrus	33	32080.6	37582.9	47.5	−0.5	−19.5
R. Frontal Pole	26	29501.4	30099.0	22.5	42	45.5
				20	42	45.5
R. Inferior Temporal Gyrus, temporooccipital part.	26	30619.0	32395.0	50	−48	−22
L. Postcentral Gyrus	22	32988.9	34580.7	−7.5	−43	55.5
R. Frontal Operculum Cortex	21	30185.1	31266.4	45	17	5.5
R. Intracalcarine Cortex	18	30436.1	32872.7	20	−60.5	5.5
R. Inferior Frontal Gyrus, pars opercularis	16	29745.9	31055.5	47.5	17	8
R. Pos. Parahippocampal Gyrus	14	29469.3	30880.5	12.5	−35.5	−7
R. Supracalcarine Cortex	14	30170.0	32475.1	22.5	−65.5	20.5
				17.5	−65.5	18
R. Insular Cortex	11	30009.5	30867.3	42.5	17.	−4.5
				40	19.5	−7
R. Pos. Superior Temporal Gyrus	10	30519.0	35460.0	47.5	−8	−17

^a^97.5th percentile TFCE threshold = 27579.2, Max network TFCE = 49329.6.

**Figure 6:**
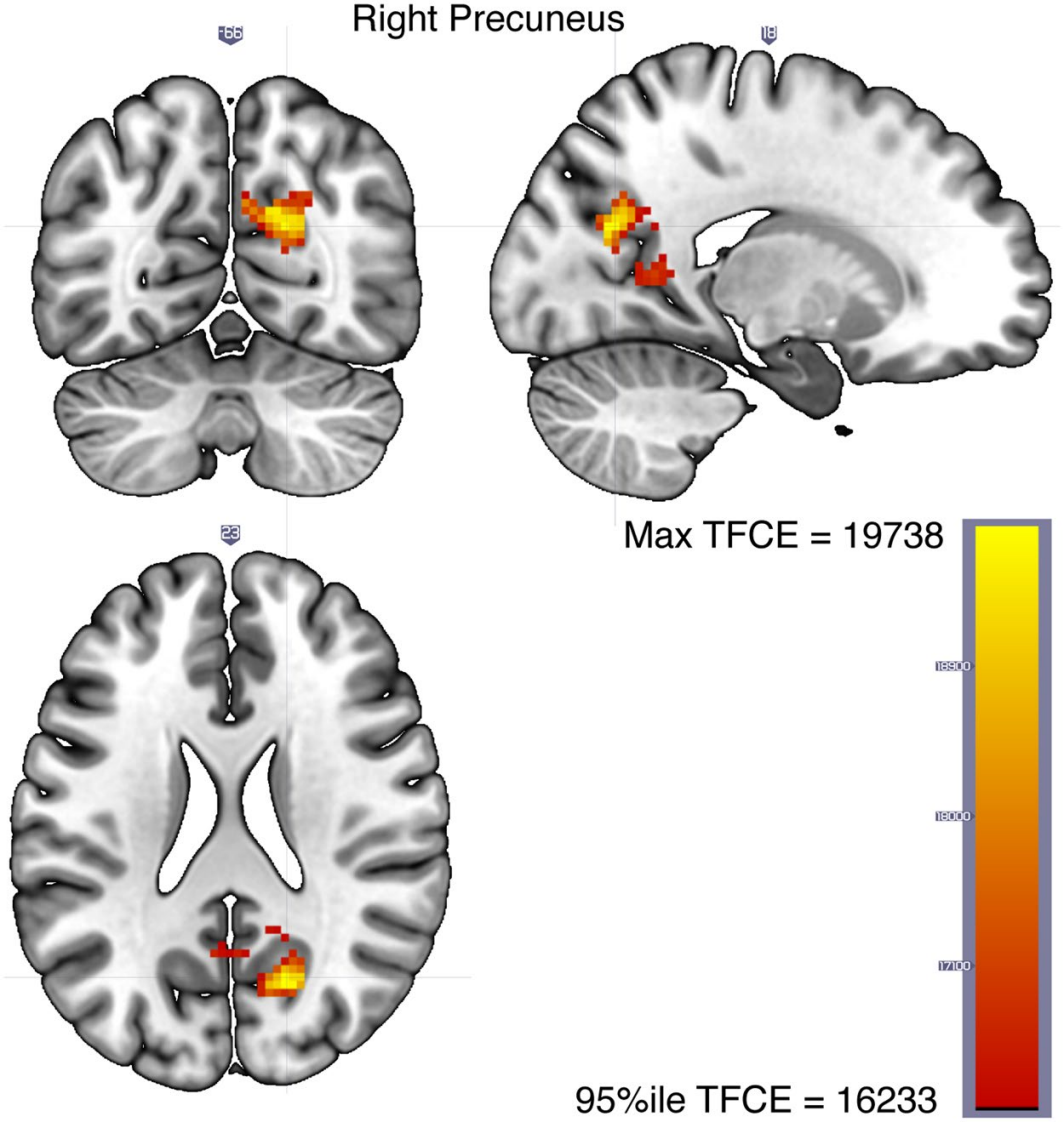
The right precuneus represents personal semantics during vivid reminiscence but to a lesser extent during non-vivid reminiscence (Eq. 6, corresponding to the conjunction between the *Hamm* and *Hamm* * *Vivid* terms, see Methods).

While the *Hamm* term in Eq. 6 tracks the regions involved in representing personal semantic content during vivid reminiscence, it does not address whether those regions distinguish between vivid and non-vivid recollection. This distinction between vivid and non-vivid reminiscence is captured by the conjunction between *Hamm* and −*Hamm* * *Vivid* which identifies regions where *Hamm* predicts neural distances for vivid pairs of images but does so to a significantly less extent for non-vivid pairs (see Methods). A dominant cluster in the right precuneus (Fig. 6) is identified as the region representing self-relevant contents of an experience when vivid autobiographical memory is generated but critically, the right precuneus content representations are significantly attenuated when the memory is non-vivid (see Table 4 for the MNI coordinates of the peak voxels in regions with at least 10 voxels in the vivid-only personal semantics network).

**Table 4:** Peak voxel coordinates of regions with at least 10 voxels in the vivid-only personal semantic network (Eq. 6, corresponding to the conjunction between the *Hamm* and *Hamm* * *Vivid* terms, also see Fig. 6).

Region	Voxel count	Mean TFCE	Max TFCE^a^	MNI coordinates		
				*x*	*y*	*z*
R. Precuneus	135	17412.7	19694.3	17.5	−65.5	23
R. Cuneal Cortex	31	17887.6	19738.4	17.5	−68	23

The FSL-Harvard-Oxford cortical-subcortical atlas was used to get coordinates in MNI space. When multiple sets of coordinates are shown for a region, they correspond to multiple peak voxels.

^a^95th percentile TFCE threshold = 16232.6, Max network TFCE = 19738.4.

**Figure 7:**
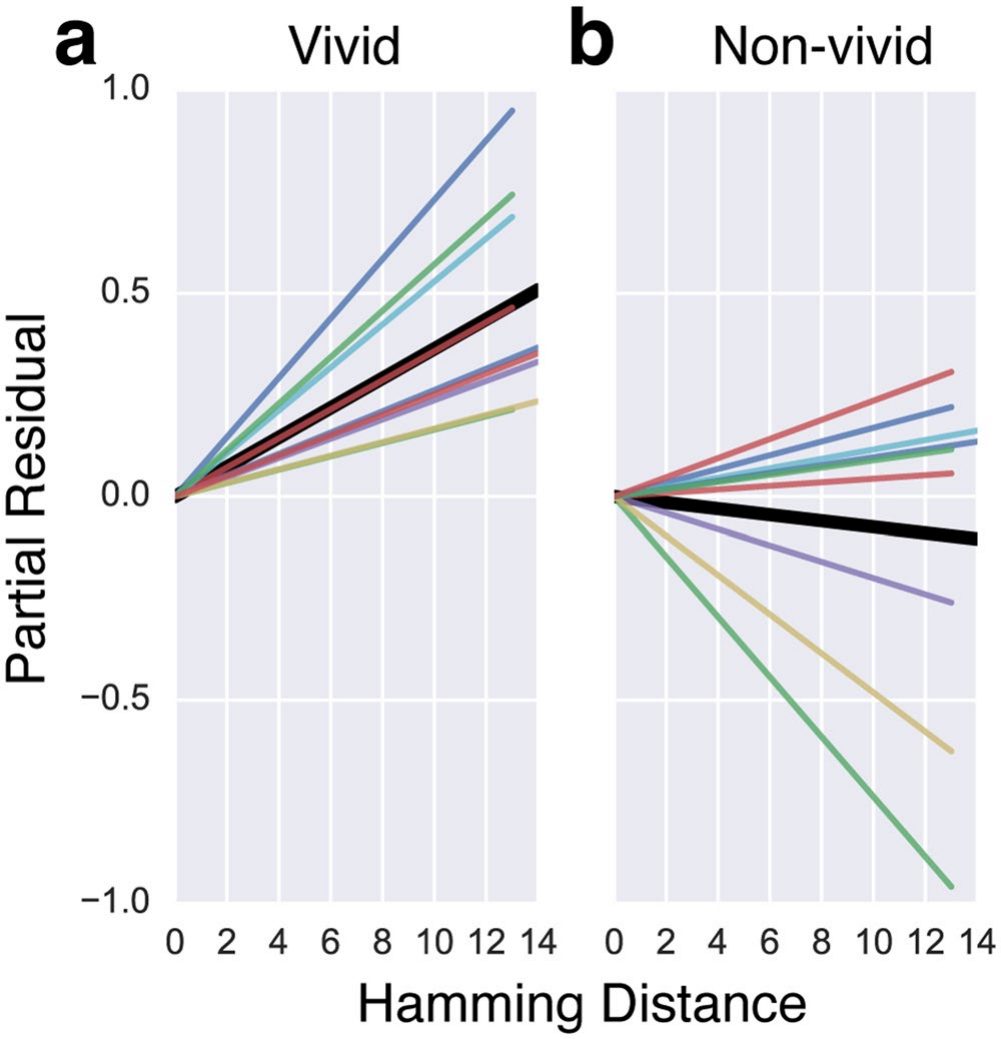
The slopes of the regression lines in Eq. 6 describing the relationship between neural distances and Hamming distances between the tag sets in a sphere of radius 7.5 mm around the peak voxel in the right precuneus. **(a)** The colored lines show individual participants’ regression lines for the relationship between Hamming distance and neural distance for vividly remembered pairs of images after accounting for the contribution from other independent variables in Eq. 6 (i.e., the partial residual). The slope of the solid black line is the mean over the individual regression lines. **(b)** The relationship between Hamming distance and neural distance for the less vividly remembered pairs of images after accounting for the contribution from other independent variables in Eq. 6. Individual participants’ plots with partial residuals overlaid are presented in Fig. 8.

**Figure 8:**
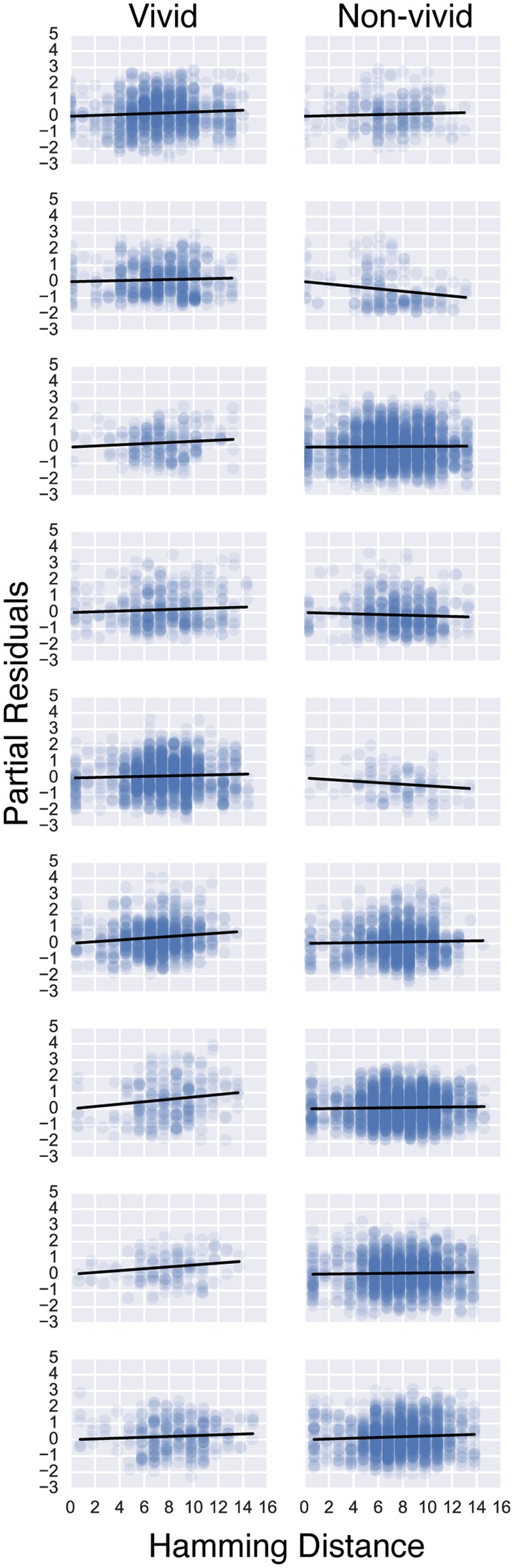
Individual participant partial residual plots of the Neural distance ~ Hamming distance relationship for vivid (left panel) and non-vivid (right panel) pairs.

Finally, we present partial residual plots to visualize the relationship between *Hamm* and neural distances in the right precuneus after taking into account the contribution from the other independent variables in Eq. 6, and we do this separately for vivid and non-vivid pairs. Since overlaying the residuals obscures the differences in the slopes of the regression lines between vivid and non-vivid conditions, we opted to display only the regression lines in Fig. 7 and the individual participants’ plots with partial residuals overlaid in Fig. 8. Figure 7a shows that neural distances in a sphere surrounding the peak right precuneus voxel are related to Hamming distances between the tag sets of vivid pairs of stimuli (*Vivid* = 0 in Eq. 6) and Fig. 7b demonstrates that this relationship is considerably attenuated for non-vivid pairs of stimuli (*Vivid* ≠ 0 in Eq. 6). These differences between vivid (more episodic) and non-vivid pairs of memories in how neural distances relate to distances between content tags suggest that vivid reminiscence is accompanied by activity in the right precuneus reflecting richer personal semantic and episodic content representations relative to non-vivid reminiscence.

In our ROI analyses, no region survived Bonferroni correction for multiple comparisons across the ROIs (used in our previous work^3^) and terms across the four models (the two models in Eqs 5 and 6 as well as the same models with space, time, and space*time interaction terms included). The regions that showed relationships between neural distances and Hamming/spatiotemporal distances (uncorrected *p* < 0.05) are presented in Table S1 in the Supplementary Information. The left anterior hippocampus represents space, time, and their interaction as reported previously^3^. The difference from the previous work is that the current models also included the Hamming distance and vividness terms. Activity in the left middle hippocampus reflected subjective content representations (across all explored models) and activity in the right posterior V1 exhibited better representations of subjective content during vivid compared to non-vivid reminiscence (conjunction analysis as described earlier, based on the models that included vividness terms, see Table S2 in the Supplementary Information). It is important to note that we did not have a specific hypothesis about the role played by these ROIs in personal semantics. The ROI analysis is presented merely as a point of comparison with our earlier work^3^.

## Discussion

In a recent review, Renoult *et al*.^6^ identified the neural correlates of personal semantics, thought to consist of facts about one’s own life extracted over many repeated experiences. They described a personal semantics network that included the medial prefrontal cortex (mPFC), retrosplenial cortex, temporal pole, posterior temporal cortex, precuneus, middle and inferior temporal gyri, inferior parietal lobe, hippocampus, parahippocampal gyrus, temporo-parietal junction, ventrolateral prefrontal cortex, and fusiform gyrus. The specific neural correlates depended on where the specific operationalization of personal semantics was located in the spectrum from semantic to episodic memory. The personal semantics network we identified in an autobiographical reminiscence task (Fig. 5a, Table 2) overlaps highly with the broad network described in Renoult *et al*.^6^ and includes core parts of the default mode network (DMN), which is thought to be involved in the processing of self-relevant information and in unconstrained mind-wandering. The DMN overlaps highly with contextual association networks and Bar *et al*.^60^ suggested that unconstrained thought processes, much like explicit associative memory processing, involve activation of such associations. Therefore, it is perhaps unsurprising that the network involved in instantiating associated personal semantic representations upon viewing an autobiographical image-cue is congruent with the associative-default network (see Fig. 1 in Bar *et al*.^61^).

We also identified a network that represented personal semantic content for vivid memories. This set of regions (Fig. 5b, Table 3) was mostly a sub-network of the more general semantic network but relatively more right lateralized. The posterior parietal cortex (including the posterior cingulate cortex (PCC) and precuneus) is a dominant part of the retrieved personal semantics networks we identified. Though studies of the human posterior parietal cortex have traditionally focused on visuospatial and sensorimotor functions, it has received increased attention recently as a region that plays an important role in episodic memory^27,34,62–64^. Though previous studies showed a predominantly left lateralized parietal retrieval network^34^, suggested that it could have been a result of the limited range of materials (mostly verbal) used in those studies (e.g. source recollection of faces vs words evokes more activity in the right hemisphere^65^, however, others have argued that retrieval MTL and posterior parietal networks are material-general^66^). Therefore, our observation of a right lateralized personal semantic network associated with vivid reminiscence could be explained by our use of highly personally relevant image cues drawn from participants’ own lives. However, since all the participants in our study were female, we are unable to rule out an alternative sex-specific explanation (*cf*.^41^) for the right lateralization (but another study^67^, with all twelve participants being female, reported a left-lateralized network in an autobiographical memory retrieval task that used verbal cues collected from family members prior to fMRI scanning).

Finally, given that vividness is a defining feature of successful autobiographical recollection, we focused on the regions within the broader network that represented retrieved personal semantic content specifically in service of vivid reminiscence, but not during non-vivid recall. The conjunction analysis identified the right precuneus as the locus of representation of content specifically accompanied by vivid reminiscence, but, critically, personal semantic representations were significantly attenuated in the right precuneus during non-vivid relative to vivid recall. Both univariate and multivariate activity in the precuneus is consistently related to vividness ratings across autobiographical memory experiments^68–73^. Furthermore, when participants are presented with family photographs, which are closer to the type of stimuli we used, univariate activity in the right precuneus and bilateral lingual gyri was associated with vividness ratings^68^. They suggested that vivid and detailed autobiographical memory was required to engage the PCC/precuneus, which is thought to represent contextual details (*cf*.^63^). Our results offer evidence for this idea by demonstrating that neural activity patterns during vivid but not during non-vivid recall in the right precuneus represent the specific self-relevant contents of the original experience as indicated by participants.

It is worth noting here that while participant-generated labels do capture personal semantic information, they also likely covary with episodic details of specific events. While we regress out the effects of visual perceptual similarity in our analysis, it remains likely that the brain regions identified represent both personal semantic and episodically-specific content, especially in the precuneus which is where representations track content labels more so for vivid memories. We take the view that these components are not all that dissociable during the course of naturalistic reminiscence and any contrived attempt to do so in the laboratory may reveal a pattern of activation that may not generalize to how people remember events in everyday life. Therefore, though we use the term “personal semantic network” to describe the set of regions identified in the analysis, we fully allow the possibility that the content labels capture aspects of autobiographical memory that lie along a spectrum between semantic and episodic, but concentrated towards the personal semantic and episodic part of that spectrum due to the highly personalized nature of the stimuli and their attributes.

The precuneus has been called the “mind’s eye”^74^ and precuneus activity is consistently associated with mental imagery and episodic memory^15,39^. A special status for the precuneus has been proposed within the default mode network^14,69,75,76^. A partial correlation-based connectivity analysis which measured the extent of interaction between nine nodes within the DMN after subtracting out the common influences from other nodes showed that precuneus was the only node that exhibited strong connectivity with virtually every other node^77^. Functional connectivity analysis^78^ and anatomical coupling and voxel-based morphometry analyses^79^ have suggested an important role for the precuneus in metacognitive ability for memory retrieval. These connectivity patterns taken together with our results suggest that the precuneus may play an important role in the integration of personal semantic information from other parts of the network leading to a detailed representation of the self-relevant contents of a specific experience, supporting the subjective experience of vivid autobiographical reminiscence.

The idea that the precuneus may have a privileged status within the DMN is further supported by the discovery that along with regions in the MTL, the precuneus is one of the first regions to be affected in early Alzheimer’s disease (AD)^80,81^. There is catastrophic breakdown of information flow when a hub in a network is affected^82^. This could explain why in the early stages of AD, people lose track of time, people, and places (also see Peer *et al*.^83^ for evidence that the same regions are important for mental orientation along the different dimensions of space, time, and persons and that the precuneus activated across these domains). On a related note, a new memory syndrome, severely deficient autobiographical memory, was identified recently^84^ in three healthy adults with otherwise normal cognitive functioning who were severely impaired on autobiographical memory function. This impairment was specific to vivid visual episodic re-experiencing of personal events but did not extend to remembering personal semantics. Furthermore, even though they were impaired relative to the controls in reporting spatiotemporally specific episodic details of remote events, they were able to produce episodic details for recent events, albeit accompanied by significantly reduced vividness ratings across all time periods. fMRI scans during a cued-autobiographical recall task revealed that there was reduced activity compared to the controls in areas including the left mPFC and right precuneus. Our results are consistent with Palombo *et al*.’s^84^ report and suggest that the subjective experience of vivid reminiscence is facilitated by activity in the right precuneus reflecting personal semantics as well as contextual details of retrieved episodes whereas personal semantics more generally are represented by a broader network of regions, which (though entirely speculative at this point) can explain the selective vividness deficits but intact personal semantics in people with severely deficient autobiographical memory.

## Conclusion

It has been suggested that AM retrieval is guided by semantic retrieval (*cf*.^8^). We identified the general network, including core parts of the default mode network, that represents retrieved personal semantics during AM search over several weeks of real-world experience. The precuneus is a hub within this network^69,77,85–87^ and our results suggest that activity in the precuneus supports the subjective experience of vivid reminiscence by representing personal semantic and subjective content attributes with greater detail during vivid compared to non-vivid recall. This account provides a plausible mechanism by which people make metacognitive judgments about their recollective experiences, and may provide key support to theories that suggest a critical role of the precuneus in the autobiograpical memory deficits seen in Alzheimer’s disease and other forms of dementia.

## Electronic supplementary material

Supplementary Movie S1

Supplementary Movie S2

Supplementary Information

## Data Availability

The datasets generated during and/or analysed during the current study are available from the corresponding author on reasonable request. The statistical maps presented in this paper are available at https://neurovault.org/collections/JBMYPSYK/. Analysis code is available at https://github.com/compmem/Sreekumar.etal.2018.
