# Supplementary material for: The experience of vivid autobiographical reminiscence is supported by subjective content representations in the precuneus
Source: Sci Rep. 2018 Oct 8;8:14899. doi: 10.1038/s41598-018-32879-0 (PMC6175904; doi:10.1038/s41598-018-32879-0)
Supplement: Supplementary file 3 — Supplementary Information [file 41598_2018_32879_MOESM3_ESM.pdf]

Supplementary Information for: The experience of vivid autobiographical reminiscence is supported by subjective content representations in the precuneus

Vishnu Sreekumar [1†], Dylan M. Nielson [1‡], Troy A. Smith [2], Simon J. Dennis [3], and Per B. Sederberg[1*]

[1] Department of Psychology, The Ohio State University, Columbus, OH, USA
[2] Department of Psychological Science, University of North Georgia, Oakwood, GA 30566
[3] School of Psychology, University of Melbourne, Melbourne, VIC, Australia
[†]Current address: Surgical Neurology Branch, NINDS, National Institutes of Health, Bethesda, MD 20892
[‡]Current address: Data Science and Sharing Team, Section on Functional Imaging Methods, Laboratory of Brain and Cognition, NIMH, National Institutes of Health, Bethesda, MD 20892
[*]Current address: Department of Psychology, University of Virginia, Charlottesville, VA 22903

[†]**Correspondence should be addressed to:**
Email: pbs5u@virginia.edu

Image Representations

Color Histogram The color histogram is a simple global image representation and is invariant under image rotation and translation. A color histogram for an image is generated by concatenating N higher order bits for features in the chosen color space. We used the Hue, Saturation and Value (HSV) space[1] since it separates color from intensity information and makes an image representation based on HSV relatively robust to changes in appearance due to differences in lighting conditions. The histogram is generated by counting the number of pixels with the same color and accumulating it in 2^{3N} bins. Quantizing the hue component more precisely than the value and saturation components makes the HSV histogram more sensitive to color differences and less sensitive to brightness and depth differences. We used a $30 \times 10 \times 3$ hue value saturation quantization of the HSV space to generate 900-dimensional color histogram image vectors.

Color Correlogram The color histogram has the drawback of being a purely global description of the color content in an image. It does not include any spatial information. Purely local properties when used can be extremely sensitive to appearance changes due to slight changes in angle, zoom, etc. Purely global properties like those used in the color histograms can give false positives in an image retrieval task as it tends to classify images from widely separated scenes as belonging to the same scene if they have similar color content. A color correlogram describes global distributions of local spatial color correlations. We followed the procedure in[2] to compute the color correlogram as follows. The color correlogram ${\gamma_{c_i,c_j}}^{(k)}$ of an image I, is a three dimensional table whose entry (c_i, c_j, k) is the probability of finding a pixel of color c_j at a distance $k \in \{1,2,3,...,d\}$ from a pixel of color c_i in the image. For pixels $p1 = (x1, y1)$ and $p2 = (x2, y2)$, we use the L_∞ norm to measure the distance between them, such that $|p1 - p2| = max(|x1 - x2|, |y1 - y2|)$. Relative to the histogram, the correlogram is robust to changes in appearance caused by occlusions, zoom, and viewing angles[3]. The size of the correlogram is $O(m^2 d)$ where m is the total number of colors and $i, j \in \{1,2,3,...,m\}$. This imposes substantial storage requirements for large values of d. So we chose to work with a compressed version of the color correlogram where we sum the conditional probabilities of color pairs over a restricted set of distances. For constructing the color correlograms, the HSV color space is quantized into $12 \times 3 \times 3$ bins. We let $k \in \{1,3,5,7\}$ and use a restricted version of the color correlogram as in Equation 1.

$$\bar{\gamma}_{c_i,c_j}(I) = \sum_{k \in \{1,3,5,7\}} \gamma^k_{c_i,c_j}(I) \tag{1}$$

This procedure resulted in 11664-dimensional feature vectors. A singular value decomposition (SVD) is carried on the 120×11664 image by feature matrix. As in our previous work[2,4,5], the decomposed image vectors are scaled by the singular values (utilizing all available 120 singular values) to generate 120-dimensional image vectors.

Histogram of Oriented Gradients (HOG) The histogram of oriented gradients (HOG) is used widely in object detection applications[6]. We used the UOCTTI variant[7] as implemented in VLFeat version 0.9.20 (`www.vlfeat.org`[8]). HOG computes a histogram of oriented gradients over square cells, typically 8 pixels per side. We also used the typical value of orientation bin size of 9. Since images were 640×480 pixels, there were 80×60 HOG cells. Dalal *et al.*[6] originally proposed normalization and truncation of HOG features via 4 normalization factors to obtain 36 HOG features. Felzenszwalb *et al.*[7] proposed alternative steps (using fewer features to speed up learning and detection) involving a principle components analysis of a collection of HOG features to derive 13 contrast-insensitive HOG features. However, their analyses also indicated that detection performance for some object classes improved when using some additional contrast-sensitive features. The end result is a 31-dimensional feature vector (see Felzenszwalb *et al.*[7] for further details). Thus, we obtain $80 \times 60 \times 31 = 148800$-dimensional HOG vectors. A singular value decomposition (SVD) is carried on the 120×148800 image by feature matrix to generate 120-dimensional image vectors.

GIST Oliva and Torralba[9] proposed a model of real-world scene recognition, based on a low dimensional scene representation that they called "Spatial Envelope". Unlike HOG, this model was not designed to detect individual objects, rather it was aimed at representing dominant spatial characteristics of a scene using a set of perceptual dimensions that were estimated using spectral and coarsely localized information. This model successfully models a holistic representation of the scene and generates a multidimensional space in which semantic categories of scenes (e.g. highways) cluster together. MATLAB code provided by the original authors was used to construct GIST representations for our analyses (`http://people.csail.mit.edu/torralba/code/spatialenvelope/`).

Speeded-up Robust Features (SURF) Speeded-up Robust Features (SURF), as the name suggests, is a fast algorithm partly inspired by the Scale Invariant Feature Transform (SIFT), that detects interest points in a view-invariant manner. We first used

the bagofFeature() function in the MATLAB computer vision system toolbox to extract SURF features for all 120 images for a given participant. As an example, 1843200 features were extracted from the image set for one participant. K-means clustering is performed to create a 500-word visual vocabulary. Each image is then represented as a histogram over these 500 clusters using the MATLAB computer vision system toolbox *encode()* function.

ROI Analysis Results

Table S1. Results from the ROI analysis. The ROIs that show a relationship between neural distances and Hamming distances between sets of tags (uncorrected $p < 0.05$) are listed in this table. The first column in the table specifies the model: "h" is the model in Equation 5 with just the $Hamm$ term (in addition to the terms that control for visual similarity and time elapsed in the scanner), "hst" is the same model enhanced with $space$, $time$, and $space * time$ terms, "hv" is the model in Equation 6 with $Hamm$, $Vivid$, and $Hamm * Vivid$ terms, and "hvst" is the same model but with additional $space$, $time$, and $space * time$ terms. The second column specifies the ROI (of the 10 we considered: anterior, middle, and posterior hippocampus, parahippocampal cortex, and posterior V1 (primary visual cortex) in both hemispheres, the third column specifies the term in the model that the result corresponds to, the fourth column provides the estimated coefficient for the corresponding term in the third column, the fifth column is the t-value for that coefficient (test across participants against 0), the sixth column is a p-value based on a permutation test, and the last column is the Bonferroni corrected p-value (correcting for 140 multiple comparisons across all ROIs and model terms) which has a ceiling of 1.000)

Model	ROI	Term	Coef	t-stat	p (perm)	p (Bonf.)
h	R. Parahippocampal Gyrus	$Hamm$	0.010	2.382	0.034	1.000
h	R. Middle Hippocampus	$Hamm$	0.012	5.083	0.002	0.280
h	R. Posterior Hippocampus	$Hamm$	0.009	2.589	0.035	1.000
h	L. Parahippocampal Gyrus	$Hamm$	0.009	2.228	0.049	1.000
h	L. Middle Hippocampus	$Hamm$	0.015	2.904	0.019	1.000
hst	R. Parahippocampal Gyrus	$Hamm$	0.010	2.310	0.036	1.000
hst	R. Middle Hippocampus	$Hamm$	0.010	4.102	0.002	0.280
hst	R. Posterior Hippocampus	$Hamm$	0.009	2.554	0.041	1.000
hst	L. Parahippocampal Gyrus	$Hamm$	0.009	2.231	0.050	1.000
hst	L. Middle Hippocampus	$Hamm$	0.0149	2.966	0.012	1.000
hst	L. Anterior Hippocampus	$space$	0.420	3.257	0.015	1.000
hst	L. Anterior Hippocampus	$space * time$	-0.064	-2.776	0.024	1.000
hst	L. Anterior Hippocampus	$time$	0.187	2.732	0.021	1.000
hv	R. Posterior Hippocampus	Viv	0.113	2.912	0.020	1.000
hv	R. Posterior V1	$Hamm$	0.022	2.650	0.029	1.000
hv	R. Posterior V1	$Hamm * Viv$	-0.018	-2.861	0.025	1.000
hv	L. Middle Hippocampus	$Hamm$	0.020	3.660	0.003	0.420
hvst	R. Posterior Hippocampus	Viv	0.116	3.000	0.019	1.000
hvst	R. Posterior V1	$Hamm$	0.023	2.642	0.034	1.000
hvst	R. Posterior V1	$Hamm * Viv$	-0.018	-3.030	0.019	1.000
hvst	L. Middle Hippocampus	$Hamm$	0.021	3.811	0.002	0.280
hvst	L. Anterior Hippocampus	$space$	0.393	3.171	0.012	1.000
hvst	L. Anterior Hippocampus	$space * time$	-0.059	-2.636	0.029	1.000
hvst	L. Anterior Hippocampus	$time$	0.173	2.621	0.024	1.000

Table S2. The ROIs that show a greater relationship between neural distances and Hamming distances between sets of tags (uncorrected $p < 0.05$) during vivid compared to non-vivid reminscence are listed in this table. The models are given by Equation 6 ("hv") as well as the same model with space, time, and space*time terms ("hvst"). The results shown here correspond to the conjunction between the $Hamm$ and $Hamm * Vivid$ terms. The t-values for $Hamm$ and $Hamm * Vivid$, and the permutation-based p-value for $\min(t_{Hamm}, -t_{Hamm*Viv})$ are displayed (only regions with uncorrected $p < 0.05$ are shown).

**Model**	**ROI**	**t-stat (Hamm)**	**t-stat (Hamm*Viv)**	**p (perm)**
hv	R. Posterior V1	2.647	-2.861	0.045
hvst	R. Posterior V1	2.642	-3.030	0.045

References

1. Smith, A. R. Color gamut transform pairs. In *Proceedings of the 5th Annual Conference on Computer Graphics and Interactive Techniques*, SIGGRAPH '78, 12–19 (ACM, New York, NY, USA, 1978). DOI 10.1145/800248.807361.
2. Sreekumar, V., Dennis, S., Doxas, I., Zhuang, Y. & Belkin, M. The Geometry and Dynamics of Lifelogs: Discovering the Organizational Principles of Human Experience. *PLOS ONE* **9**, e97166 (2014). DOI 10.1371/journal.pone.0097166.
3. Huang, J., Kumar, S. R., Mitra, M., Zhu, W.-J. & Zabih, R. Image indexing using color correlograms. In *Proceedings of IEEE Computer Society Conference on Computer Vision and Pattern Recognition*, 762–768 (1997). DOI 10.1109/CVPR.1997.609412.
4. Doxas, I., Dennis, S. & Oliver, W. L. The dimensionality of discourse. *PNAS* **107**, 4866–4871 (2010). DOI 10.1073/pnas.0908315107.
5. Sreekumar, V., Dennis, S. & Doxas, I. The Episodic Nature of Experience: A Dynamical Systems Analysis. *Cogn. Sci.* **41**, 1377–1393 (2017). DOI 10.1111/cogs.12399.
6. Dalal, N., Triggs, B. & Schmid, C. Human Detection Using Oriented Histograms of Flow and Appearance. In *Computer Vision – ECCV 2006*, Lecture Notes in Computer Science, 428–441 (Springer, Berlin, Heidelberg, 2006). DOI 10.1007/11744047_33.
7. Felzenszwalb, P. F., Girshick, R. B., McAllester, D. & Ramanan, D. Object Detection with Discriminatively Trained Part-Based Models. *IEEE Transactions on Pattern Analysis Mach. Intell.* **32**, 1627–1645 (2010). DOI 10.1109/TPAMI.2009.167.
8. Vedaldi, A. & Fulkerson, B. Vlfeat: An Open and Portable Library of Computer Vision Algorithms. In *Proceedings of the 18th ACM International Conference on Multimedia*, MM '10, 1469–1472 (ACM, New York, NY, USA, 2010). DOI 10.1145/1873951.1874249.
9. Oliva, A. & Torralba, A. Modeling the Shape of the Scene: A Holistic Representation of the Spatial Envelope. *Int. J. Comput. Vis.* **42**, 145–175 (2001). DOI 10.1023/A:1011139631724.
